# Subacute vessel wall imaging at 7-T MRI in post-thrombectomy stroke patients

**DOI:** 10.1007/s00234-019-02242-9

**Published:** 2019-06-25

**Authors:** My Truong, Karin Markenroth Bloch, Mads Andersen, Gunnar Andsberg, Johannes Töger, Johan Wassélius

**Affiliations:** 1 Medical Imaging Department, Neuroradiology, Skåne University Hospital, 22185 Lund, Sweden; 2 Department of Clinical Sciences, Lund University, Lund, Sweden; 3 Lund University Bioimaging Center, Lund University, Lund, Sweden; 4 Philips Healthcare, Copenhagen, Denmark; 5 Department of Neurology, Skåne University Hospital, Lund, Sweden; 6 Diagnostic Radiology, Department of Clinical Sciences, Skåne University Hospital Lund, Lund University, Lund, Sweden

**Keywords:** 7-T MRI, Vessel wall imaging, Thrombectomy, Embolic stroke, Stent retriever

## Abstract

**Purpose:**

Reports from 3-T vessel wall MRI imaging have shown contrast enhancement following thrombectomy for acute stroke, suggesting potential intimal damage. Comparisons have shown higher SNR and more lesions detected by vessel wall imaging when using 7 T compared with 3 T. The aim of this study was to investigate the vessel walls after stent retriever thrombectomy using high-resolution vessel wall imaging at 7 T.

**Methods:**

Seven patients with acute stroke caused by occlusion of the distal internal carotid artery (T-occlusion), or proximal medial cerebral artery, and treated by stent retriever thrombectomy with complete recanalization were included and examined by 7-T MRI within 2 days. The MRI protocol included a high-resolution black blood sequence with prospective motion correction (iMOCO), acquired before and after contrast injection. Flow measurements were performed in the treated and untreated M1 segments.

**Results:**

All subjects completed the MRI examination. Image quality was independently rated as excellent by two neuroradiologists for all cases, and the level of motion artifacts did not impair diagnostic quality, despite severe motion in some cases. Contrast enhancement correlated with the deployment location of the stent retrievers. Flow data showed complete restoration of flow after treatment.

**Conclusion:**

Vessel wall imaging with prospective motion correction can be performed in patients following thrombectomy with excellent imaging quality at 7 T. We show that vessel wall contrast enhancement is the normal post-operative state and corresponds to the deployment location of the stent retriever.

**Electronic supplementary material:**

The online version of this article (10.1007/s00234-019-02242-9) contains supplementary material, which is available to authorized users.

## Introduction

Stroke is the second cause of mortality worldwide, the second cause of dementia, and the most common cause of adult disability [1]. Approximately, 80% of all strokes are ischemic [2], i.e., caused by thromboembolic occlusion of a vessel. Following several randomized controlled trials showing the superiority of endovascular thrombectomy over solely intravenous thrombolysis, endovascular thrombectomy has been established as the standard of care for acute stroke with large vessel occlusion [3–7].

Computed tomography (CT) and magnetic resonance imaging (MRI) are the imaging modalities used for diagnosis, MRI being the most sensitive and specific method capable of diagnosing very early ischemia. There is accumulating evidence that vessel wall imaging of intracranial vessels can be readily performed with MRI at field strengths of 3 and 7 T [8–13]. MRI is also capable of quantifying blood flow in individual vessels within the circle of Willis [14].

Recent MRI studies using vessel wall imaging at 3-T field strength have shown thickening and gadolinium contrast enhancement in the vessel wall following acute stroke treatment (intravenous thrombolysis and endovascular thrombectomy), for large artery occlusion [15, 16]. It is also known that endovascular procedures such as aneurysm coiling can result in vessel wall contrast enhancement, assumed to be caused by mechanical damage to the endothelium from guidewires and catheters [17].

Using 7-T instead of 3-T field strength results in an increased signal-to-noise ratio (SNR) and allows for image acquisitions with higher resolution. This is also true when using low-SNR imaging techniques such as black blood vessel wall imaging. The higher resolution makes it possible to visualize non-diseased vessel walls without the use of a contrast medium. However, to be able to see this level of detail, minimal patient motion is required during the scan. This can be challenging for patients recovering from severe stroke, especially considering the relatively long scan times required. To counter patient motion, there is a newly developed prospective motion correction technique that can be applied to the vessel wall sequence, but this has not yet been performed in many studies [18, 19].

The aim of the study was to confirm the findings from previous studies using MRI at 3-T field strength by using 7 T, and also determine whether the observed vessel wall abnormalities were due to the lodged thrombus or to the mechanical trauma from the devices used to extract the thrombus. Furthermore, we wanted to explore the use of motion correction for high-field vessel wall MRI to compensate for patient movements during the examination.

## Material and methods

### Patients

Patients (*n* = 7, mean age 69 years, range 55–84 years) treated with endovascular thrombectomy for acute stroke in the anterior circulation with a CTA-verified thrombus in the distal internal carotid artery (T-occlusion), or M1- or M2-segments of the middle cerebral artery from February 2018 to June 2018 were included in the study. Details of the patient cohort are listed in Table 1.

**Table 1:** Patient data including age, sex, affected side, NIHSS (National Institute of Health Stroke Score) before treatment and at 7-T MRI examination, the treated vessel segment (*M* middle cerebral artery, *T* distal carotid “T-occlusion”), TICI-score, the diameter and length of the stent retriever used

Patient	Age	Sex (M/F)	Affected hemisphere	NIHSS pre-treatment	NIHSS at MRI	Treated vessel	Number of attempts	TICI score	Stent retriever width/length (mm)
1	80	F	R	15	3	M1	1	2B	4/40
2	69	F	R	9	2	M1	2	2B	5/33
3	61	F	L	5	0	M2	1	2B	5/33
4	84	F	R	3	1	M2	1	3	5/33
5	74	F	L	20	3	M2	1	3	4/40
6	55	M	R	8	1	M1	1	3	4/40
7	67	M	R	20	6	T + M1	1	3	4/40

Exclusion criteria included the inability to give informed consent, MR safety concerns at 7 T, or expected inability to complete the examination, for example due to cognitive impairments such as neglect. Seven patients in total were included, and all examinations were performed without sedation.

The study was approved by the local research ethics committee and written informed consent was obtained from all patients prior to participating in the study.

### Endovascular thrombectomy procedure

The endovascular procedures were performed via a 9 French femoral artery access. Site of occlusion was confirmed by digital subtraction angiography (DSA) from a balloon guide catheter in the internal carotid artery. The occlusion was crossed by microguidewire and microcatheter. Passage beyond the occlusion was confirmed by DSA. A stent retriever was deployed covering the thrombus, typically with its most proximal part, and retracted after a 5-min delay during balloon occlusion of the internal carotid artery and continuous aspiration in the guide catheter. Table 1 includes the specific stent retrievers used. Treatment result was assessed by repeated DSA from the internal carotid artery.

### 7-T MRI protocol

MRI was performed within 2 days of the thrombectomy using an actively shielded 7-T MRI unit (Philips Achieva, Best, The Netherlands) with a dual channel transmit, 32 channels receive, head coil (Nova Medical, Wilmington, MA, USA). Dielectric pads were placed over the temporal lobes in order to increase the transmit efficiency in these areas [20] (Leiden University Medical Center, Leiden, The Netherlands). Pulse oximetry was used for cardiac gating in flow scans.

The imaging protocol included a 3D T1 weighted fast gradient echo sequence for the planning of flow measurements (3D TFE, 1 × 1 × 1 mm^3^ resolution, duration 1:40 min), through-plane flow measurements in the right and left M1-segments (0.5 × 0.5 × 3 mm^3^ resolution, duration 1:35 min per segment), and vessel wall imaging using a 3D magnetization prepared inversion recovery turbo spin echo sequence (MPIR-TSE, 0.8 × 0.8 × 0.8 mm^3^ resolution, duration 10:32 min). Sequence details are reported in Supplemental Table 1. The 3D MPIR-TSE, described in detail by van der Kolk et al. [11, 21] is a volumetric black blood sequence with an inversion pulse to null CSF and magnetization preparation to increase SNR. This was performed prior to gadolinium contrast administration (Gadoteric acid 0.2 ml/kg bodyweight) and post-contrast administration. The post-contrast 3D MPIR-TSE was started at least 3 min after the contrast injection.

To reduce motion artifacts which cause blurring of the vessel walls, a prospective motion correction using a newly developed navigator framework (iMOCO) [18, 19] was applied to the 3D MPIR-TSE sequence. For quantifying motion, fat-selective 3D gradient echo navigator volumes [22] were inserted in the time gaps after each TSE readout in the 3D MPIR-TSE sequence. Each reconstructed navigator volume was compared with the first volume in real-time, and the position and angulation of the MPIR-TSE volume was correspondingly updated before the next repetition of the MPIR-TSE. Furthermore, a motion score combining translation and rotation was calculated [23], and if the detected motion was larger than a certain threshold (1 mm in this protocol), the last shot of the MPIR-TSE was reacquired using the updated geometry settings. Reacquisition prolongs the scan duration but reduces motion artifacts. The iMOCO technique displays the detected motion on the operator console during acquisition, allowing the operator to monitor the amount of motion and reacquisitions throughout the scan.

### Image analysis

Image analysis was performed independently by two neuroradiologists with over 5-year experience (MT and JW). The analysis was undertaken at least 3 months after the MRI examination to minimize the risk of recall bias.

Image assessment was initially performed on the vessel wall images where the vessel wall thickening was noted on the pre-Gd images. The gadolinium contrast uptake was noted on the post-Gd images and the length of the contrast-enhanced segment was measured on curved multiplanar reconstructions. The circumferential distribution of the enhancement/thickening was noted. The location of any Gd uptake was correlated to the location of the embolus based on the pre-operative CT angiography, and the location of the stent retriever based on the per-operative DSA images. The image analysis is illustrated in Supplemental Fig. 1.

The degree of motion artifacts was graded according to a pre-defined scale, and overall image quality in both contrast phases (pre-Gd and post-Gd) was graded according to another pre-defined scale. Other relevant findings such as vessel occlusions, ischemic lesions, or hemorrhage were also noted (not reported here).

Flow data were analyzed using Segment v2.2 R6324 (http://segment.heiberg.se) [24]. After performing a linear background correction, quantitative values on flow, flow rates, and velocities were obtained.

## Results

**Fig. 1:**
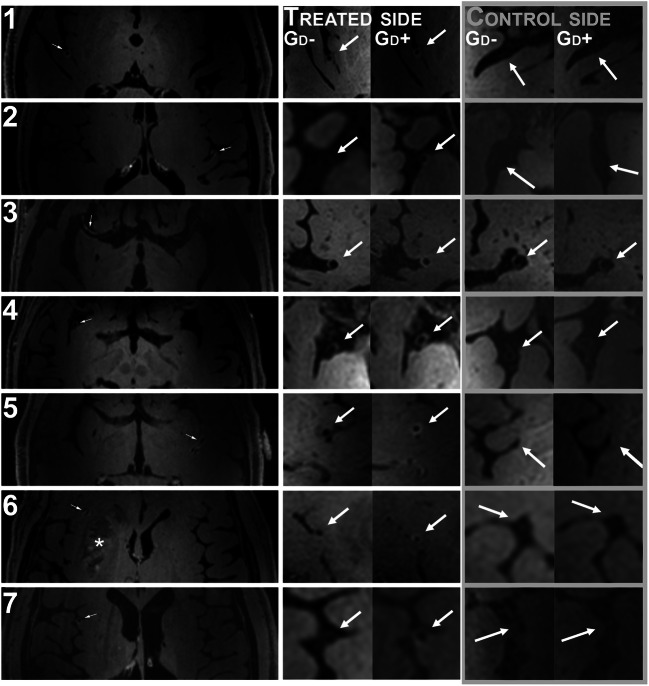
Transaxial images post-Gd images from all 7 subjects showing the Gd uptake in the vessel walls at a location corresponding to the position of the stent retriever (arrows in left and middle columns), with absence of Gd uptake on the contralateral side (right column). The asterisk indicates a basal ganglia infarct in patient 6. All images in the left column were reconstructed from sagittal acquisitions with meticulous adjustments to obtain exact transaxial reconstructions to allow comparison with the contralateral side. The images in the middle and right columns were reconstructed to obtain image planes perpendicular to the center-line to illustrate the circumferential distribution of vessel wall edema pre-Gd (middle column Gd−) and the post-Gd enhancement (middle column Gd+). On the contralateral side (right column), the vessel vas often barely visible, and in cases where the vessel was occasionally thicker, suggesting intracranial atherosclerosis, little or no Gd-uptake was seen (illustrated in patient 3/control Gd− and Gd+)

**Fig. 2:**
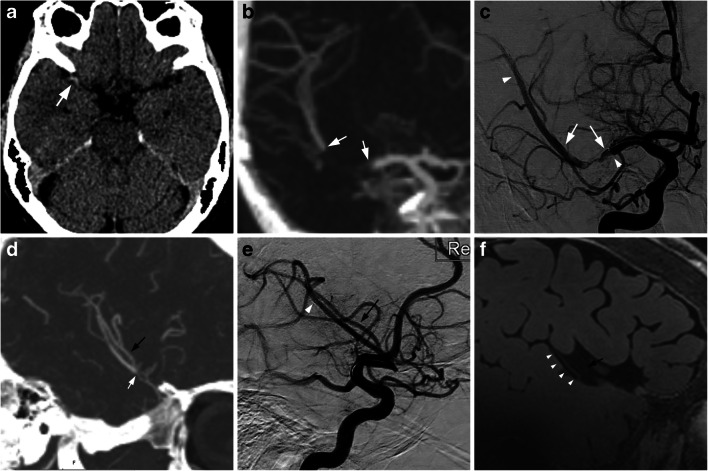
**a** Illustration of the typical findings in one of the patients. The site of the embolus was determined from the dens artery sign on non-enhanced CT (white arrow), **b** occlusion on CTA (white arrows indicating the proximal and distal end of the embolus), and **c** DSA images from the thrombectomy procedure (white arrows indicating the proximal and distal end of the embolus, white arrowhead indicating the distal end of the stent retriever). The dominant M2 branch distal of the embolus in which the stent retriever was deployed (the white arrow in panel D indicates the distal end of the embolus, the white arrowhead in panel E indicating the distal end of the stent retriever) shows vessel wall Gd uptake corresponding to the entire length of the stent retriever (white arrowheads in panel F). **d**–**f** The black arrows illustrate another M2 branch distal of the occlusion that shows no vessel wall Gd uptake

In general, all patients tolerated the examination well. For one patient, there was significant motion during the post-contrast MPIR-TSE, causing the scan to be aborted and reacquired. Figure 1 shows transaxial images from the post-Gd vessel wall 3D MPIR-TSE from all seven patients and images perpendicular to the center line of the vessel pre- and post-Gd to illustrate the typical circumferential vessel wall thickening and contrast enhancement that was continuous for several centimeters, corresponding to the deployment of the stent retriever. Figure 2 illustrates one case in more detail, including the relation between the embolus, the stent retriever, and the vessel wall Gd uptake.

The results of the image assessment by the two neuroradiologists are shown in Tables 2 and 3. Both reviewers identified vessel wall Gd uptake on the side that had been treated during the thrombectomy, and in all cases both reviewers agreed that the Gd uptake correlated to the location of the stent retriever rather than to the site of the embolus (Table 2). The agreement between reviewers was excellent.

**Table 2:** The results of the MRI imaging assessment by the two blinded reviewers (reviewer 1/reviewer 2) for the side of vessel wall gadolinium contrast (Gd) uptake, correlation with the location of the embolus, the stent retriever or both, overall image quality on the pre- and post-contrast acquisitions, the amount of motion artifacts in the post-contrast acquisitions, and presence of large vessel occlusions

Patient	Gd uptake side (R/L)	Gd uptake correlation (embolus, stent retriever or both)	Image quality pre-Gd (1–3)*	Image quality post-Gd (1–3)*	Motion artifacts (1–3)^✝^	Large vessel occlusion (yes/no)
1	R/R	Stent/stent	3/3	3/3	1/1	No/no
3	L/L	Stent/stent	3/3	3/3	2/2	No/no
4	R/R	Stent/stent	3/3	3/3	2/1	No/no
2	R/R	Stent/stent	3/3	3/3	2/1	No/no
5	L/L	Stent/stent	3/3	3/3	2/1	No/no
6	R/R	Stent/stent	3/3	3/3	2/2	No/no
7	R/R	Stent/stent	3/3	3/3	2/2	No/no

^✝^Level of motion artifacts: 1 = none, 2 = visible but not affecting image quality, 3 = impairing image quality

*Overall image quality: 1 = non-diagnostic, 2 = acceptable, 3 = excellent

**Table 3:** Image analysis data including hyperintensity previous to gadolinium contrast administration on the affected side (ipsilateral) and the contralateral side, wall thickness with/without contrast, any visible vessel wall edema on ipsilateral side/contralateral side, and the length of vessel wall enhancement

Patient	Hyper-intensity pre-Gd ipsilateral	Hyper-intensity pre-Gd contra-lateral	Wall thickness pre-Gd ipsilateral (mm)	Wall thickness post-Gd ipsilateral (mm)	Wall edema ipsilateral side	Wall edema contra-lateral side	Length of wall enhancement (mm)
1	No	No	0.7–1.30	1.3–1.6	Yes	No	53
2	No	No	0.7–0.8	1.0–1.2	Yes	No	86
3	No	No	0.7–1.0	0.9–1.3	Yes	No	51
4	No	No	0.5–0.9	0.8–1.2	Yes	No	62
5	No	No	0.7	1.1–1.4	No	No	45
6	No	No	1.0	1.0–1.4	No	No	32
7	No	No	0.8	1.3–1.4	No	No	53

Overall image quality was graded highest on a 3-grade scale (1 = non-diagnostic, 2 = acceptable, 3 = excellent) for all patients in all contrast phases, by both reviewers.

Motion artifacts were rated as *none* or *not affecting diagnostic quality* for all examinations by both reviewers. Neither reviewer rated the motion artifacts as *impairing the diagnostic quality* for any case.

**Fig. 3:**
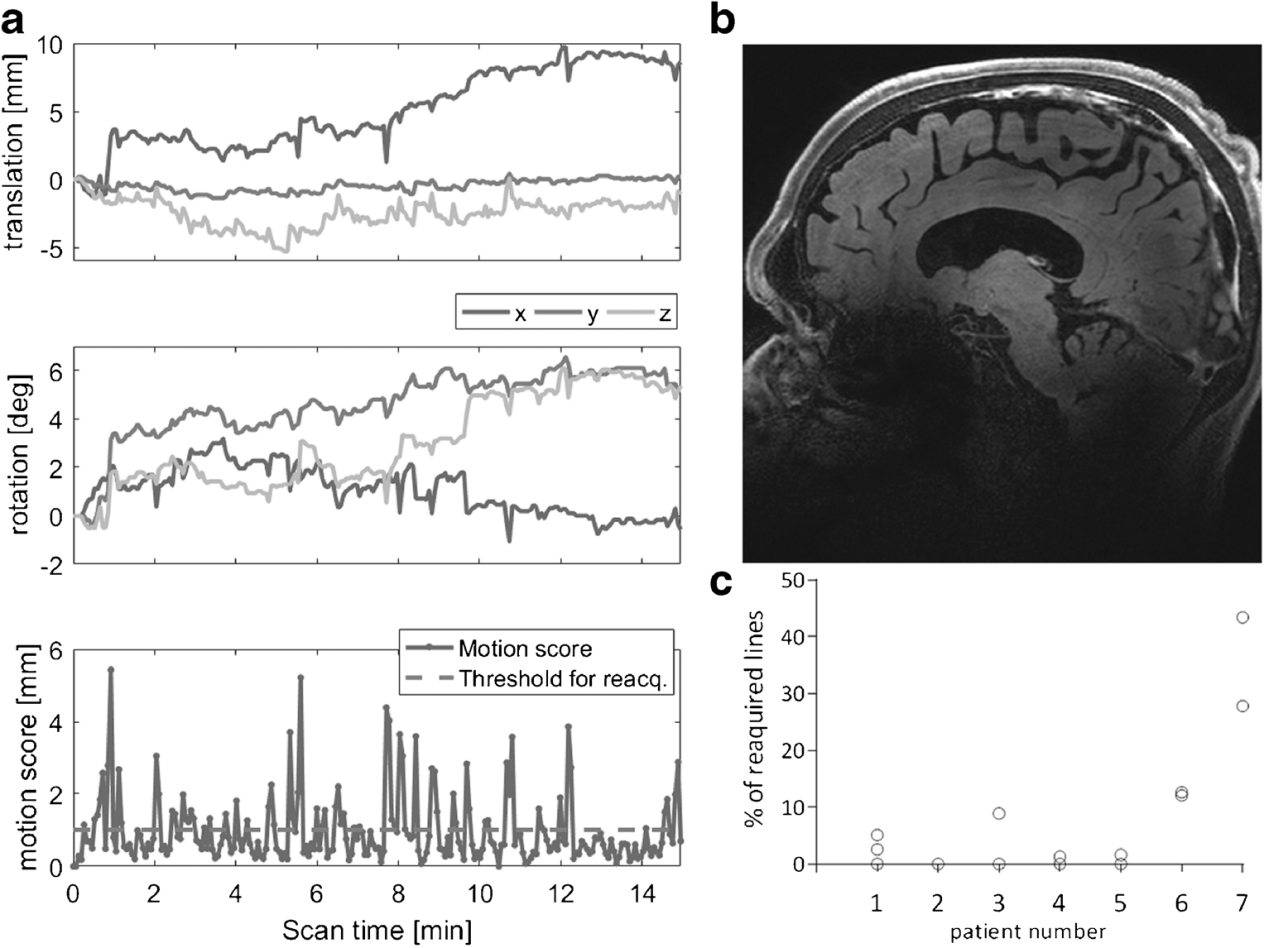
Effects of prospective motion correction. The monitored motion during the post-Gd acquisition for the patient that moved the most (patient 7) (**a**). The translations (top left panel) shows the translation in millimeters in x, y and z rotations (middle left panel) shows the position relative to the start in degrees. During the scan, the motion score (bottom left panel) is continuously monitored. In this case, the patient moved almost 1 cm, and rotated up to 6°. Motion larger than the chosen threshold (dashed line in the lower left panel, set to 1 mm in our protocol) triggers reacquisition of the previously acquired k-space segment. Despite this severe motion, the image quality was excellent as shown by an image from the same acquisition (**b**). The percentage of reacquired data for all 14 vessel wall scans, clearly showing that the movements in patient 6 and 7 demanded reacquisition of a large part of the data (**c**)

In total, 14 MPIR-TSE sequences were acquired. In eight of these, the motion never exceeded the reacquisition threshold, so the scan time was unaffected. In the remaining six MPIR-TSE scans, the percentage of reacquired data was between 1.6 and 43% (median 11%), which corresponds to a scan prolongation of between 10 s and 3:39 min, respectively (median 61 s). Figure 3 a shows the motion observed for patient 7. The patient moved gradually almost 1 cm in the x direction and rotated 5–6° around *y* and *z* axes during the scan. Motion larger than the threshold (dashed line in the lower panel in Fig. 3a) triggered reacquisition of the last shot. Figure 3 c displays the amount of reacquired data for all 14 vessel wall scans, clearly showing that the movements in patients 6 and 7 demanded a reacquisition of a large part of the data. Despite this severe motion, the image quality was excellent (Fig. 1, patients 6 and 7, Table 2 and Fig. 3b).

**Fig. 4:**
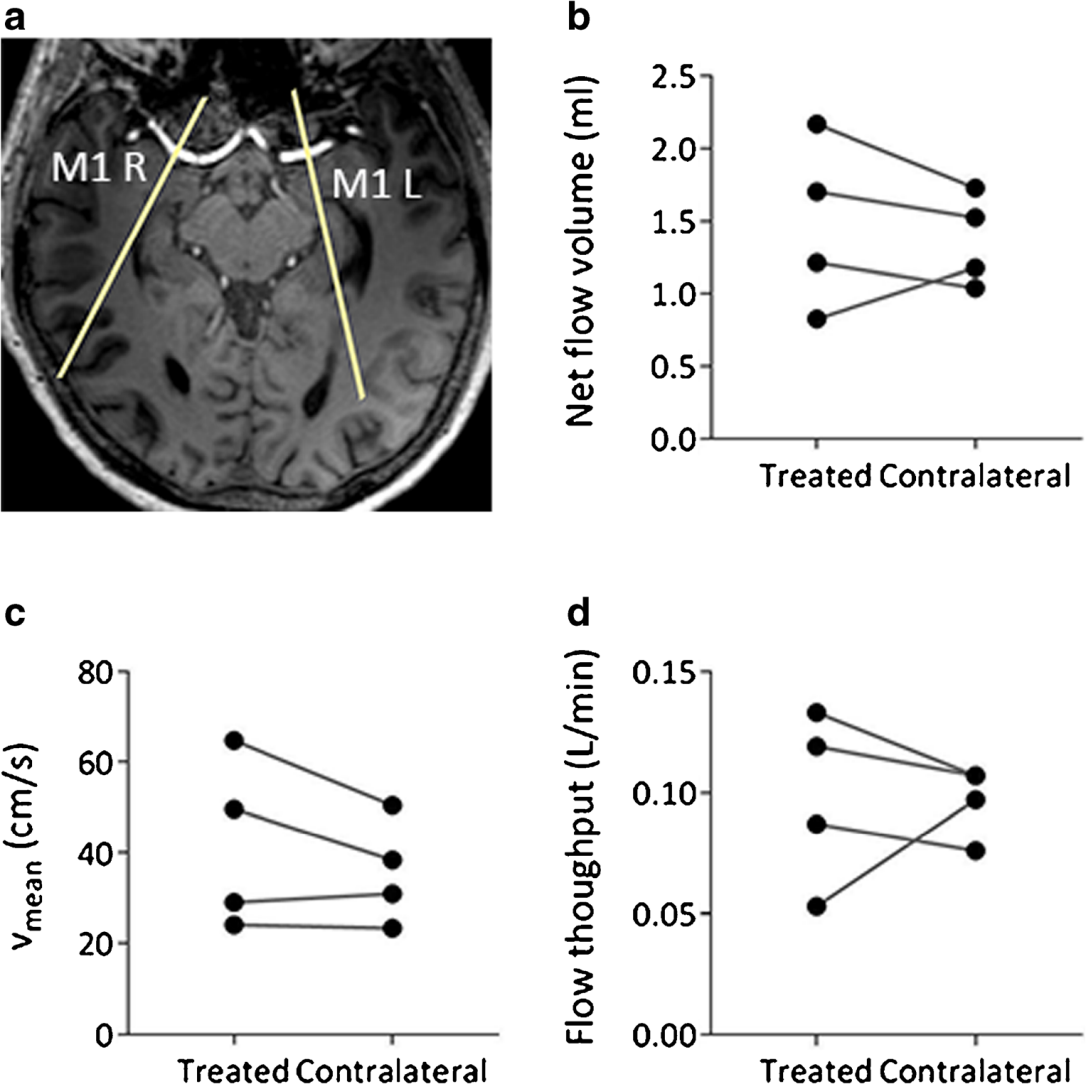
Measurements in the MCA M1 branch on the treated and contralateral side (*N* = 4). **a** The placement of the flow measurement planes, **b** the net flow volume (ml/heart beat), **c** the average velocity (cm/s), and **d** total flow (L/min). There is no significant difference in the average values for any variable, but in this small sample there is a trend towards a larger spread of values in the treated vessel

In three of the patients, the cardiac triggering was unreliable, and no flow data were acquired. Flow data in both M1 segments were obtained in the remaining four patients. There were no differences between the treated and untreated vessel, either in average values of net flow per heartbeat, mean velocity, or flow throughput per minute (Fig. 4).

## Discussion

This prospective study was performed to confirm previously reported findings of changes in the vessel wall following endovascular thrombectomy using MRI at 7-T field strength, and to assess whether the vessel wall contrast enhancement correlates to the location of the thrombus or the stent retrievers. Furthermore, we wanted to study the use of motion correction for high-field vessel wall MRI to compensate for patient movements.

We now report a consecutive series of 7-T vessel wall imaging following successful stent retriever thrombectomy. The application of prospective motion correction in the sub-acute phase yielded excellent diagnostic images without significant motion artifacts.

Previous vessel wall imaging studies have mostly been performed on mixed treatment populations, whereas our population received the same acute stroke treatment. Power et al. reported definite vessel wall enhancement in four out of six patients treated with stent retriever thrombectomy, with possible enhancement in the remaining two [15]. However, Seo et al. reported vessel wall enhancement in only five out of nine patients treated with single pass stent retriever thrombectomy [16]. In the present study, we found consistent vessel wall enhancement in all seven patients, possibly owing to the increased signal-to-noise ratio at 7 T compared with 3 T, and the use of prospective motion correction to mitigate motion artifacts. We also established that vessel wall enhancement in all cases correlated with the deployment zone of the stent retriever rather than the location of the lodged embolus. It is known that endovascular procedures on intracranial blood vessels, such as aneurysm coiling, can result in vessel wall contrast enhancement [17], and mechanical interference to the vessel intima during stent retriever thrombectomy could potentially be extensive, since the over-sized stent is deployed and retracted along extensive parts of the affected vessel. Thus, vessel wall contrast enhancement seems to be the normal state following stent retriever thrombectomy, and we can visualise it better at 7-T MRI with prospective motion correction compared to conventional 3T MRI.

Recently, specialized black blood sequences at 7-T MR have been shown to yield high resolution vessel wall images [8, 11, 12, 21, 25]. Direct comparisons demonstrate that 7-T images reveal more vessel wall lesions than a 3-T examination, and that the vessel walls also display a higher contrast to the surrounding tissue [10]. Validations of wall thickness measurements have been performed by comparing MRI imaging of ex vivo specimens with histological measurements [26].

In our series of seven patients, image quality was independently rated excellent by two experienced neuroradiologists, and with the use of a novel prospective motion correction technique (iMOCO), neither of the reviewers noted that motion artifacts impaired the diagnostic quality, despite severe motion in some cases. This is in contrast to the study by Harteveld et al. [10] where prospective motion correction was not used, and motion artifacts hampered assessment in several cases.

It was not possible to obtain quantitative flow data in three of the subjects due to unreliable cardiac triggering. For the four patients where flow data were obtained, no significant differences in flow parameters could be seen between the treated and untreated sides, which is expected with a complete restoration of normal blood supply.

This study is limited by the small number of subjects, and future studies with larger study groups are warranted to support our findings. Furthermore, longitudinal studies would be desirable to determine how long vessel wall enhancement is sustained.

## Conclusions

We conclude that 7-T vessel wall imaging with prospective motion correction in the sub-acute phase following endovascular thrombectomy is safe, robust, and reliably yields excellent diagnostic images. The present study clearly shows that vessel wall Gd uptake is the normal post-operative state following stent retriever thrombectomy and corresponds with the deployment zone of the stent retriever. Prospective motion correction was useful in mitigating motion artifacts even though some patients moved considerably during examination. Hence, high-resolution vessel wall imaging at 7 T with prospective motion correction is a useful tool for future studies on the effects of interventions on vessel walls.

## Electronic supplementary material

Supplemental Table 1 MRI sequence parameters (DOCX 16 kb)

Supplemental Figure 1 Illustration of the image analysis process. Panels 1 and 5 illustrate the reconstructed curved multi planar reconstruction used to measure. Panel 2 illustrates how the vessel wall thickness/enhancement was measured post-Gd. Panel 3 illustrates an image plane perpendicular to the center-line to illustrate the circumferential distribution of vessel wall edema pre-Gd and the post-Gd showing enhancement in panel 4. (PNG 343 kb)

High Resolution Image (TIF 3357 kb)

## Abbreviations

MRI: Magnetic resonance imaging
Gd: Gadolinium
VW: Vessel wall
ICA: Internal carotid artery
MCA: Medial cerebral artery
M1: First segment of the medial cerebral artery
M2: Second segment of the medial cerebral artery
TICI score: Thrombolysis in cerebral infarction score
NIHSS: National Institutes of Health Stroke Scale
