# Supplementary material for: Subacute vessel wall imaging at 7-T MRI in post-thrombectomy stroke patients
Source: Neuroradiology. 2019 Jun 25;61(10):1145–53. doi: 10.1007/s00234-019-02242-9 (PMC6754352; doi:10.1007/s00234-019-02242-9)
Supplement: Supplementary file 1 — MRI sequence parameters (DOCX 16 kb) [file 234_2019_2242_MOESM1_ESM.docx]

**Supplemental Table 1 MRI sequence parameters**

|  | 3D TFE^1^ | 3D MPIR-TSE^2^ | 3D Fat Navigator | 2D flow |
| --- | --- | --- | --- | --- |
| Acquired in-plane resolution (mm x mm) | 1x1 | 0.8x0.8 | 7x7 | 0.5x0.5 |
| Reconstructed in-plane resolution (mm x mm) | 1x1 | 0.5x0.5 | 5.2x5.2 | 0.5x0.5 |
| Slice thickness | 1 | 0.8 | 7 | 3 |
| Number of slices | 190 | 238 | 21 | 1 |
| Repetition time, TR (ms) | 5 | 3952 | 3 | 15 |
| Echo time, TE (ms) | 2 | 38 | 1.38 | 4.8 |
| α° | 6 | 68 | 4 (binomial, fat-selective) | 7 |
| Echo train length^3^ | 450 | 156 | - | - |
| Temporal segmentation factor | - | - | - | 2 |
| Inversion time, TI (ms) | - | 1375 | - | - |
| Velocity sensitivity, Venc (cm/s) | - | - | - | 200 |
| SENSE factor | 2x2 | 2x3 | 2x2 | 2 |
| Partial Fourier factor | - | - | 0.85x0.85 | - |
| Dynamic scan time (ms) | - | - | 484 | - |
| Time duration (min: s) | 1:37 | 10:32 | - | 1:35 |

1. Turbo Field Echo
2. Magnetization Prepared Inversion Recovery-Turbo Spin Echo
3. For TFE: Turbo Field Echo factor, for TSE: echo train length
