# Supplementary figures and images for: Subacute vessel wall imaging at 7-T MRI in post-thrombectomy stroke patients
Source: Neuroradiology. 2019 Jun 25;61(10):1145–53. doi: 10.1007/s00234-019-02242-9 (PMC6754352; doi:10.1007/s00234-019-02242-9)

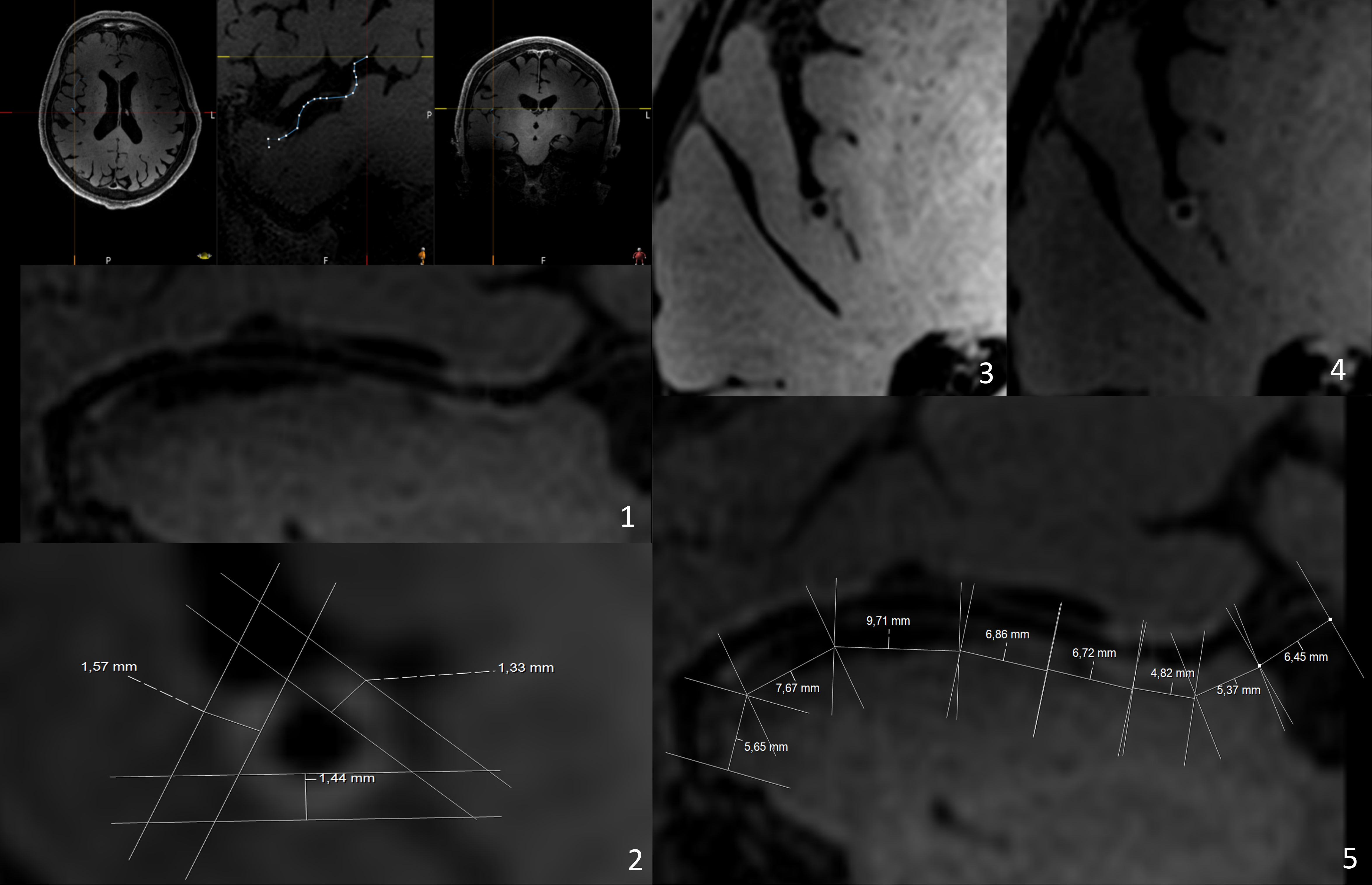

Supplement: Supplementary file 2 — Illustration of the image analysis process. Panels 1 and 5 illustrate the reconstructed curved multi planar reconstruction used to measure. Panel 2 illustrates how the vessel wall thickness/enhancement was measured post-Gd. Panel 3 illustrates an image plane perpendicular to the center-line to illustrate the circumferential distribution of vessel wall edema pre-Gd and the post-Gd showing enhancement in panel 4. (PNG 343 kb) [file 234_2019_2242_Fig5_ESM.png]
